# Feasibility of Physical Therapy Evaluation Symptom Provocation Tests in Older Adults With Mild Traumatic Brain Injury: Mixed Methods Study

**DOI:** 10.2196/76799

**Published:** 2025-10-23

**Authors:** Carrie A Barrett, Mark G Goetting, Alessander Danna-dos-Santos

**Affiliations:** 1 Department of Physical Therapy, College of Health and Human Services, Western Michigan University, 1903 W. Michigan Ave., Kalamazoo, MI, 49008, United States, 1 2693878369; 2 Department of Pediatric and Adolescent Medicine, Western Michigan University Homer Stryker School of Medicine, Kalamazoo, MI, United States

**Keywords:** older adults, geriatric, mild traumatic brain injury, concussion, physical therapy evaluation, symptom provocation tests

## Abstract

**Background:**

Early standard assessment protocols have decreased costs and better identified treatment strategies in individuals with mild traumatic brain injury (mTBI). Clinical practice guidelines contained strong recommendations for the use of provocation tests in evaluating younger adults (<65 y of age). Currently, the recommended protocols and literature regarding tolerance to self-reported and physical exertion outcome testing in older adults (>65 y of age) with mTBI have been limited. To start bridging some of these shortcomings and aid the development of practice guidelines for older adults, this study explored the feasibility of physical therapy evaluation protocols for individuals aged >65 years.

**Objective:**

The study aimed to (1) assess the feasibility and tolerance of using evidence-based physical therapy evaluation outcome tests, and (2) apply the International Classification of Health, Disability, and Function (ICF) domains to an emergent thematic analysis of research protocols involving older adults with and without mTBI.

**Methods:**

The feasibility study was a mixed methods design that included 13 community participants (>65 y) with and without mTBI. Investigators documented completion of the health form, participant-reported outcomes including the Patient-Specific Function Scale (PSFS) and Post Concussion Symptom Scale, and physical performance measures using the Motion Sensitivity Quotient (MSQ) and a submaximal recumbent stepper test, modified from the Buffalo Concussion Bike Test. Emergent contextual themes were identified within the study protocol, testing space, and participant responses.

**Results:**

The sample included 13 participants (aged 65‐91 y; 7 females, 6 males), 4 with mTBI and 9 without mTBI. All completed the health form, PSFS, and Post Concussion Symptom Scale, with moderate verbal cues required in 15% (PSFS) of the cases. The Motion Sensitivity Quotient and Buffalo Concussion Bike Test-Modified were completed to the participants’ maximum safe effort. No participants experienced adverse mTBI symptoms. The categories for the theme of study protocol were time, communication, and logistics coordination. The categories for the theme of study environment were assessment, documentation, and safety. Unanticipated positive emotions were evoked with comments such as feedback, including participation was “fun” and “I didn’t know I could do all this.”

**Conclusions:**

Older adults were able to tolerate the collection of participant-reported and physical outcome measures that allowed for individual maximal safe efforts without mTBI symptom provocation. The contextual themes using the ICF model of study protocol and environment were categorized and coded for future research consideration. Positive emotion participant responses were captured at the end of data collection. Clinical Relevance: This study applied standard physical therapy assessment protocols, which were safely tolerated by older adults with mTBI. The study used ICF domains from a research procedure perspective for protocol and environment considerations. The findings included participants’ positive emoted responses, which may inform future large-sample trials in the evaluation of older adults with mTBI.

## Introduction

Researchers reported over 4.3 million individuals with traumatic brain injury (TBI) annually in the United States [1]. Of all TBI severities, experts considered 90% to be “mild” traumatic brain injury (mTBI) [2]. However, it is important to note that despite the “mild” designation, individuals with mTBI developed cognitive, physical, psychological, and social dysfunctions [3,4]. Of importance, persisting symptoms posed a higher risk to older adult populations (aged >65 y) [3-6]. Investigators have reported that 34% (all ages) have mTBI symptoms after 6 months, whereas over 44% of older adult populations continued to have symptoms beyond 6 months [3,6]. Researchers described ongoing mTBI symptoms to include headache, irritability or emotional lability, sleep disruption, drowsiness or fogginess, dizziness, and forgetfulness [7].

The aging population of older adults with mTBI has negatively contributed to the US health care burden [4,8,9]. Researchers reported the overall health care cost of those with mTBI was US $46 million (2017) [8]. Pavlov et al [9] found that health care costs were 2 times higher in older adults than in individuals in younger populations among those with mTBI. Older adults with mTBI reported having difficulty in self-care, mobility, and quality of life [4]. Due to the complex clinical scenario resulting from mTBI, researchers reported a pressing need for the development of clinical assessment tools that effectively assess the extent of symptoms and use intervention strategies [10,11].

Research has supported that early standard assessment or evaluation protocols may help decrease costs and better identify treatment strategies for individuals with mTBI [11,12]. Clinical practice guidelines for physical therapists contained strong recommendations for the use of test measures in those who have persistent symptoms after mTBI in younger populations [7,13]. The recommended tests included patient positional testing and aerobic testing, which mildly provoked mTBI symptoms [13]. These tests provided the necessary information to initiate rehabilitative treatment [13,14]. Unfortunately, the provocation testing in mTBI research failed to include findings on older adult populations [11,13]. The purpose of this study was to explore the tolerance to the recommended testing in a small group of older adults with and without mTBI. The study aims were (1) to determine the feasibility and tolerance in older adults in implementing the recommended outcome tests for mTBI, and (2) to describe an emergent thematic analysis during the testing of older adults with and without mTBI within the domains of the International Classification of Health, Disability, and Function (ICF) model. This study has detailed the methodology and participant responses to provide preliminary considerations in older adult research. Further development of physical therapy evaluation guidelines for these tests will guide clinicians in safe, evidence-informed assessment of the aging population with mTBI [7,11].

## Methods

### Recruitment

The feasibility study was a mixed methods design conducted at Western Michigan University (WMU) Laboratory for Advancements in Rehabilitation Sciences. The participants were recruited from local clinics, hospital emergency departments, senior living facilities, and senior centers. A total of 15 community members were interested in the study. Of the members not enrolled (n=2), 1 expressed severe physical limitations prohibiting travel to the study site, and 1 declined due to the data collection schedule. The investigators established the sample size based on the timeline of funding and successfully enrolled 13 eligible older adults (June 2022- June 2023).

The inclusion criteria included individuals aged >65 years, who had the cognitive means to answer health-related questionnaires and the physical ability to perform testing. The general cognitive and physical abilities were screened via verbal communication over the telephone during the study explanation. In this screening, the primary investigator (PI) explained the study protocol and asked participants if they had concerns about memory testing or exercising on a stationary stepper. The PI (CAB) assessed for verbal inconsistencies in language, memory, and processing throughout all interactions with participants. In addition, the PI (CAB) screened the cardiovascular system, the cervical spine and neck, and mobility status of each participant to determine medical clearance and safety before data collection. The exclusion criteria excluded ages younger than 65 years; screening abnormal for neck screening (ligamentous instability or vertebral artery insufficiency) [13]; or not having the physical ability to perform the aerobic testing [15].

The PI (CAB) collecting data was state-licensed, certified in advanced older adult cognitive screening and cardiopulmonary resuscitation (basic life support), and worked as a board-certified specialist in neurologic physical therapy for 17 years in direct access, providing services to patients with neurologic conditions.

### Ethical Considerations

The WMU institutional review board (IRB) reviewed and approved this study under the expedited review with a waiver of written informed consent (application IRB-2022‐205). The participants were assigned study numbers, and all data were collected on hard copy forms. The data were entered into the computer as deidentified data. The raw data were analyzed under the study numbers. The coinvestigators (AD-d-S, MGG, Lizzie Butkis, Ashley Helmer, and Mike Peres) analyzed all deidentified data. All participants verbalized and signed the written IRB-approved consent before enrollment. The study was grant-funded by the WMU Faculty Research and Creative Award (grant 2020). Through this funding, the participants received US $30 gift card incentive to participate in the study.

### Study Design

Research supported that older adults with mTBI could complete participant-reported and health status forms [3,5]. Previous studies included individuals with mTBI experiencing symptom provocation when they were exposed to visual, cognitive, and movement stimulation [16,17]. Investigators (CAB, Ashley Helmer, Mike Peres, and Lizzie Butkis) created an a priori observational table to document the completion of each participant-reported form. There was limited research on a similar tool. The senior coinvestigators (AD-d-S and MGG) peer-reviewed the observation table. After the collaboration, all investigators unanimously agreed to mark the completeness of each of the forms to be marked as “completed without or minimal assistance,” “completed with moderate assistance,” and “unable to complete: [reason].” Participants were categorized as “completed without or with minimal assistance” when they independently marked all form prompts without or with less than 50% cues provided by the investigator (verbal, visual, and physical) to complete missed form prompts. Participants were marked as “completed with moderate assistance” when they needed cues more than 50% and up to 100% of the time in filling out each form. Participants were considered “unable to complete: [reason]” when they were not able to complete the form, even with visual, verbal, or physical cues from the PI, and the reason reported. The “unable to complete” was anticipated to be used for participants who became overstimulated from significant mTBI symptoms due to cognitive or physical processing demands.

### Statistical Analysis

#### Feasibility

The first focus of this study was to explore the participants’ tolerance and ability to complete physical therapy evaluation testing in older adults with and without mTBI. The PI (CAB) was to monitor and record the participants’ responses, tolerance, and vitals during the participant-reported and physical measurements. The participant-reported measures included a general health form, the Post Concussion Symptom Scale (PCSS) [18], and the Patient Specific Functional Scale (PSFS) [13,19,20]. The PI (CAB) collected data regarding participant abilities (including field notes on impairments of mobility, communication, and hearing) and responses to filling out the forms. The PI (CAB) recorded the physical measures of the Motion Sensitivity Quotient (MSQ) [21], and the Buffalo Concussion Bike Test-Modified (BCBT-M) [14,15,22] to determine mTBI sign or symptom provocation and safe participation. A recumbent stepper with adjustable arm handles, leg pedals, and a supportive seat was used as a modification to the Buffalo Bike Concussion Test to accommodate a variety of abilities in older adult participants for aerobic submaximal physical exertion testing [23,24]. Previous research has supported the validity and reliability of the test measures and parameters collected (Table 1). The PI (CAB) recorded observations on the participant’s ability to safely complete each health medical form, PCSS, PSFS, MSQ, and BCBT-M; therefore, we used descriptive statistics for analysis.

**Table 1.** Data collection measurement summary feasibility study in older adults with mild traumatic brain injury.

Measure	ICF^a^ domains	How measured	Variable type	Psychometrics of geriatric or mTBI^b^ outcomes
Health form and screening	Body structure function - physical screen	Participant-reported and investigator	Binary (yes or no)	Clinical practice guidelines [13]
PCSS^c^	Body structure function - symptom limitation	Participant-reported	Numeric (0‐132)	Responsive rate (effect size and standardized response mean>1.3) [18]
PSFS^d^	Activity participation	Participant-reported	Numeric (0‐10)	Reliability and validity (92% completed, β=.477, *P*=.02) [19,20]
Completion of participant-reported forms (PCSS, PSFS)	Tolerance to cognitive exertion	Investigator	Categorical (completed without or minimal assist, completed with moderate assist, not completed [reason])	—^e^
MSQ^f^	Body structure function, activity - screen of vestibular, cervical spine, motion sensitivity, benign paroxysmal positional vertigo, and balance	Investigator	Numeric (0‐20)	Reliability (ICC^g^ 1.00‐0.95) and validity (*r*=0.64, *P*<.001, Cronbach α=0.95) [21]
BCBT-M^h^	Body structure function - exertional tolerance or aerobic capacity	Investigator	Numeric (blood pressure, heart rate, relative perceived exertion, and symptom provocation intensity)	Reliability (95% CI <0.01-0.03, *P*=.03) and validity (*z*=−2.5, IQR 16-20; *P*=.013) [15,22,25]
Completion of physical performance (MSQ, BCBT-M)	Tolerance to movement or physical exertion	Investigator	Binary (yes or no)	—

a ICF: International Classification of Function, Disability, and Health.

b mTBI: mild traumatic brain injury.

c PCSS: Post Concussion Symptom Scale.

d PSFS: Patient Specific Functional Scale.

e Not applicable.

f MSQ: Motion Sensitivity Quotient.

g ICC: intraclass correlation coefficient.

h BCBT-M: Buffalo Concussion Bike Test-Modified.

#### Study Themes

The secondary focus of this study was to describe emergent themes [26], or concepts that investigators recorded during the study that could impact the data collection and findings in older adults with and without mTBI. In patient-care settings, physical therapists commonly use the World Health Organization–developed ICF model to capture a comprehensive understanding of the patient [27]. Conversely, researchers have not commonly used the ICF model for research procedures or setup. We determined that contextual domains of the ICF model (environment and personal), that commonly have been integrated into a clinical physical therapy appointment, were not captured in the data collection of this study. Therefore, we created a thematic analysis early in the study to explore similar contextual factors from a research perspective [26]. We defined the contextual factors as the interaction of the environment and study protocol among older adults with and without mTBI. After all data were collected and deidentified, we (CAB, Ashley Helmer, Mike Peres, and Lizzie Butkis) reviewed the data to derive categories and codes. We met 1-2 times a week via videoconference call or electronically, to develop common observations among all participants, and all investigators met to determine final agreement on terminology.

The 2 major themes were identified as the study protocol and study environment. We defined the study protocol as an outlined plan for conducting the research that included considerations of methodological processes, participant contextual details (participant ability and interaction during the task), and factors related to recording data that may be specific to older adult research. The study protocol categories included (1) time, or a unit of measure needed to complete a task or action; (2) communication, or an informational exchange that included an understanding; and (3) logistics, or coordination of person, place, and task. The study environment was defined as the physical space and the investigator’s interaction with older adult participants before, during, and after the data collection. The study environment theme included (1) assessment, or the action of making a judgment of the participant’s ability; (2) documentation, or accurate recording of information as evidence; and (3) safety, or mitigation of risk of harm or injury (Table 2). The PI (CAB) documented the observations of the study protocol and test environment, and the coinvestigators (MGG, AD-d-S, Ashley Helmer, Mike Peres, and Lizzie Butkis) iteratively derived emergent themes, categories, codes, and examples based on deidentified written responses. The PI (CAB) performed member checking after each recorded participant’s comment by repeating the comment back to the participant to ensure accuracy. The code of “unsolicited participant feedback post data collection” emerged as a single content area that the senior investigators agreed to further explore as important (MGG, AD-d-S, Ashley Helmer, Mike Peres, and Lizzie Butkis). The PI (CAB) recorded these unanticipated responses from participants as field notes. The PI (CAB) captured the quotes and repeated them back, or member checked, to ensure accuracy. The quotes were later analyzed (MGG, AD-d-S, Ashley Helmer, Mike Peres, and Lizzie Butkis) for contextual relevance. In previous studies, researchers described contextual relevance as a nuanced understanding, tailored to a specific population within a given event or circumstance [28].

**Table 2.** Emergent thematic analysis of data from a feasibility study in older adults with mild traumatic brain injury: study protocol and study environment (n=13).

Themes (categories)	Codes	Examples (subject number) (P^a^ and or I^b^) impact
Study protocol (time, communication, and logistics)	Participant contact, study explanation, and scheduling calls	Telephone communication, “I’m having a hard time hearing you” (1,3) (P and I) “I have another call and will be putting you on hold/call you back” (4,6,7) (P and I) Participant leaving a message and their perception of not calling back is a returned call 2 d after the message (4,6,13) (P) “Can you repeat what I’ll be doing?” (4,13) (P and I) “I’ll need a ride”. “because I can’t drive” (1,3,13,10) (P)
Study protocol (time, communication, and logistics)	Delivery of study instructions to participants for the day of the study	“Can you mail them to me? I don’t use e-mail” (5,6,13) (I)
Study protocol (time, communication, and logistics)	After testing, participant recovery	Assess for independent mobility, or cool down required for more than 10 min after testing (3,5,9,13) (I)
Study protocol (time) Study environment (documentation, assessment, and safety)	Physical assist vehicle to or from the data collection site	Due to way-finding and participant requests, PI^c^ met participants in the parking lot and walked them to the test site (1-12) (I) Due to slight abnormal gait after testing, the participant was assisted to their vehicle (3,5,7,12,13) (I) Poor straight path walking to and from the test room observed (5,7,12) (I) After testing observed to have postexercise coughing upon leaving the test space (9,13) (P and I)
Study environment (assessment) Study protocol (time and logistics)	Individual testing, arriving with partner	Couples signed up or needed to ride together (3,4,6,8,11,12) (P and I)
Study protocol (time and communication)	Review of consent before data collection	Time needs to adjust PI speech for response (speak louder, lower, or bias to the left or right ear), slow speech pace for the participant to understand, and provide verbal space for participants with a delayed response (9,10,12) (I)
Study protocol (time and communication) Study environment (documentation, assessment, and safety)	Data collection	Social discussion by participants during (1-13) (I) Repeat instructions, 1-word cues, and time for the participant to move and process requested task (9,10,12) (I) Assessment of participant tolerance, safety (1-13) (I) Skill in providing safety in the use of gait belt, assistive device, when to stop testing in RPE^d^ and BP^e^ or HR^f^ critical ranges (1-13) (I)
Study environment (documentation, assessment, and safety)	Equipment use (stationary recumbent stepper, exam table, gait belt, BP stethoscope, cuff, and HR monitor)	Aerobic equipment modification for UE^g^ or LE^h^ usage and position modifications (ability, joint safety, comfort) (1-13) (P and I) Determine variant size BP cuff (10,13) (I) Use of gait belt when observed compensations, slight veer, or reach for furniture in balance or mobility (5,7,10,12) (I) Document safety considerations, modifications of equipment (1-13) (I) Document responses to exertion while observing and assisting the participant (1-13) (I)
Study protocol (time and logistics) Study environment (assessment and safety)	Lab space (standard chair without wheels, fan, water, bathroom nearby)	“I can’t sit in a roller chair - I’ll never get up” (3,12) (I) Observed difficulty getting up from a chair (3,5,12) (I) “It’s hot”, observed to need to cool off-fan (9,13) (I) Request to use the bathroom during data collection (5,8,11) (I) Request of bathroom close to the data collection room (2,5,8,9,12,13) (P) Request of water for medication or thirst during data collection (1,4,9,12,13) (I)
Study protocol (time and communication) Study environment (documentation and assessment)	Unsolicited participant feedback post data collection	Language included in the study was “fun” (1,6,7,11) (P) “I didn’t know what to expect, and I did o.k.” (5,9) (P) “I didn’t know I could do all this” (2) (P) Language that requested participation in “the next study”. (5,6,7,12) (P) “Is that it? I thought it would take longer!” (3) (P) “I really liked this” (2) (P) “I’m happy to help out” (7) (P) Assess to walk unassisted, “I’m fine to walk to my car by myself, I feel really good” (13) (P)

a P: participant impact to consider during the study.

b I: investigator impact to consider for study set-up.

c PI: primary investigator.

d RPE: relative perceived exertion (0=best, 10=worst).

e BP: blood pressure.

f HR: heart rate.

g UE: upper extremity.

h LE: lower extremity.

## Results

### Feasibility of Completion

The total sample was 13 (n=9 without mTBI, n=4 with mTBI), including participants aged 65‐91 years (n=7 female). The participants reported their race to be White (n=10) or chose not to fill out race (n=3). The participants fully completed the health form without or with minimal assistance (N=13, 100%). Most participants fully completed the PSFS (n=11, 85%) with minimal to no cues. The participants who completed the PSFS with moderate verbal cues (n=2, 15%; n=1 without mTBI, n=1 with mTBI) required verbal cues to define “activities” and redirection due to their extensive details of chosen activities. All the participants completed the PCSS (N=13, 100%) with minimal to no cues. The participants completed the MSQ and BCBT-M to their maximum safe effort. In the MSQ, 1 participant with mTBI tested positive for benign paroxysmal positional vertigo (BPPV) and needed 50% assistance to complete testing. A different participant without mTBI, in completing the MSQ, required 75%‐90% assistance for the standing and turning component due to a previous diagnosis of bilateral lower extremity neuropathy. Most of the participants (n=10, 77%) were not able to fully complete the 10 minutes of the BCBT-M due to contraindicated cardiovascular pulmonary responses or reported joint discomfort. Of importance, no participants experienced significant adverse mTBI signs or symptoms from any of the testing [15].

### Emergent Themes

The participants’ and the investigators’ perspectives were observed within the identified themes of study protocol and environment. For example, the investigators described the theme of the study protocol of time, as the time needed for the participant to communicate, transfer, walk, and write; and the time allowed by the investigator to allow space to complete the tasks (Table 2). Similarly, the investigators defined the theme for the study environment to include categories of assessment, documentation, and safety from each of the participants and the investigator’s perspectives. For example, documentation influenced the participant’s ability to complete participant-reported forms (denoted by P, participant impact) and the investigator’s ability to record data while concurrently monitoring the participant (designated by I, investigator consideration). The codes under only the study protocol theme included “patient contact, explaining the study, scheduling calls, and study site visit” which related to telephone usage; “delivery of patient test day instructions” which revealed a need to communicate through general postal mailing as email was not an option; “explaining the consent slowly allowing space to ask questions,” which increased the timeframe of data collection; and “after testing participant recovery,” which required time, skilled assessment, and directions to guide the participant to safely cool down. The code under only the study environment theme was “equipment use.” Various participant and investigator interactions and perspectives contributed to the overlapping of the categories, codes, and examples in both the study protocol and environment themes (Table 2). The codes that included both themes were physical assistance and testing, data collection, laboratory space, and participant feedback.

### Contextual Relevance

**Figure 1.**
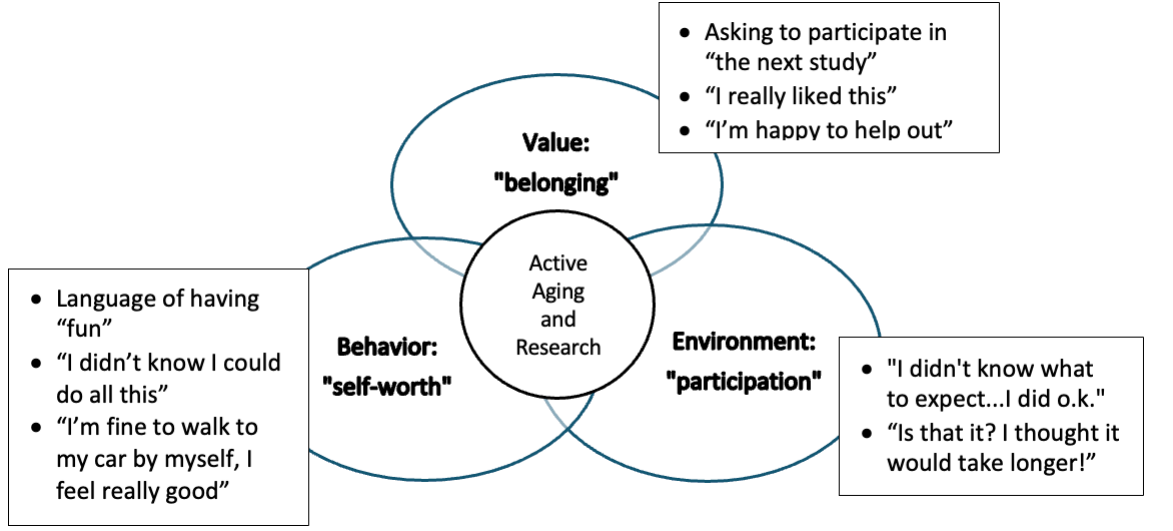
Role theory of active aging in a feasibility study with corresponding older adult participant feedback (the Role Theory concept was adapted from the works of Ding and Ran [29] and Anglin et al [30]).

The contextual relevance findings were further analyzed due to the unanticipated positive emotions evoked by the participants [28]. In the code “unsolicited participant feedback,” participants stated comments such as, “that was fun,” and “I didn’t know I could do all this.” In the analysis of the participants’ quotes, we conceptualized the participants’ perceptions of their roles and how they identified in society through the framework, the Role Theory of Active Aging (Figure 1) [29]. According to researchers who described the Role Theory of Active Aging, as individuals age, their perceived societal roles change, as does a potential loss of identity and a lack of understanding of their new roles [29,30]. The three main contributing categories that intersect within the Role Theory of Active Aging included (1) value or positive social role recognition, (2) environment or the space that allows for role fulfillment, and (3) behavior or interaction based on role expectation. Respectively, under these categories, this study applied the Role Theory of Active Aging concepts of (1) self-worth or a term for one’s internal sense of being worthy of or good enough for a task, person, or situation; (2) belonging or one’s affinity of inclusion, acceptance, or identity; and (3) participation or the action of taking part in an action or event (Figure 1). The concepts all overlapped, thereby contributing to active aging [29]. We adapted this framework to active aging through older adult participant research involvement.

## Discussion

### Overview

Previous research has supported standard evaluation and assessment protocols for individuals aged <65 years with mTBI, including medical history interviews, self-reported outcomes, motion provocation screening, and submaximal aerobic testing [11,13,25]. The research supporting mTBI management in older adults has been limited [11]. This study provided early findings that the use of standard evaluation measures was safe in participants aged >65 years. This feasibility study included two aims: (1) to determine the feasibility of older adults’ tolerance to the mTBI practice guidelines with a focus on participant-reported forms and symptom provocation tests, and (2) to describe an emergent thematic analysis contextualized within the ICF model relevant to research protocols and data collection in aging populations.

### Principal Results

#### Tolerance to Provocation Testing

The first aim was to determine the feasibility of older adults tolerating participant-reported and physical outcomes of mTBI testing. These preliminary findings supported that older adults (aged 65‐91 y) were able to complete the participant-reported outcomes of the medical health form (N=13, 100%), the PCSS (N=13, 100%), and the PSFS (n=11, 85%) without or with minimal assistance (Table 3). A small subset of participants required assistance to complete the PSFS (n=2, 15%). Assistance was related to defining the activity and providing redirection to remain on task (n=1 without mTBI, n=1 with mTBI). None of the participants reported post-mTBI symptoms from the task of completing these forms. These findings of completing the PSFS were consistent with previous findings in the use of the PSFS in older adult populations and individuals with acquired brain injuries [19,20]. In addition, these findings were also consistent with previous research in symptom-reported measures on individuals with complete and incomplete recovery after mTBI [31] and activity limitation subjective forms in individuals with acquired brain injury [19]. This study supported that older adults were able to complete standard forms that provided information about mTBI history, symptom intensity, and functional impact. The information from the forms has been crucial in guiding clinicians throughout the examination and development of early management strategies [13]. These preliminary findings additionally supported that older adults were able to complete the physical measures of MSQ (n=12, 92%) and the BCBT-M (n=13, 100%) without significant adverse effects. Quatman-Yates et al [13] reported that more research was needed to determine the outcome utility of motion-provoking symptom testing after mTBI. Researchers have supported that the MSQ outcome was reliable and valid in screening for symptom provocation related to the vestibular system, cervical spine, BPPV, motion sensitivity, and balance, which differentiated impairments and guided treatments [13,21]. This study’s preliminary findings supported that the older adults with and without mTBI tolerated the MSQ testing and further identified 1 participant who was positive for BPPV. Our early findings in the BCBT-M were consistent with the research on the Buffalo Concussion Treadmill Test and the Buffalo Concussion Bike Test in the use of submaximal aerobic testing in individuals with mTBI; however, we modified the aerobic equipment to a recumbent stationary stepper to allow for the flexibility of appropriate fitting in this older adult population [23,24]. Importantly, all the participants tolerated the stationary recumbent stepper testing (n=13), completing the BCBT-M to their safest optimal level without provocation of mTBI symptoms. We anticipated the participants with mTBI to have exertional symptom provocation, and the results did not indicate this, possibly due to the small sample size. Most of the participants (n=10, 77%) discontinued the test before the standard 10-minute mark due to suboptimal cardiovascular responses or musculoskeletal discomfort during movement. These findings suggested the standard submaximal exertion test protocols designed for populations aged <65 years may not be the same for individuals >65 years with or without mTBI. Participants reported cardiovascular-altering medication usage during data collection. We are in the process of comparing participant medication use, early test termination due to poor cardiovascular responses, and participant perception of exertion to inform future research. Previous studies have described the need to develop BCBT-M protocols for older adults with and without mTBI, and although not generalizable, this study informed tolerance to BCBT-M in older adults [13,15]. The preliminary data in this study also indicated aerobic limitations attributable to musculoskeletal and deconditioning and did not indicate adverse or unsafe mTBI symptom responses. Similar exertional testing in individuals younger than 65 years with mTBI has been used to establish performance levels and to define aerobic exercise parameters that support brain health and accelerate healing after mTBI [13,15]. The development of age-appropriate parameters in older adults is essential to ensure safe and effective rehabilitation following mTBI.

**Table 3.** Results of feasibility in testing older adults with mild traumatic brain injury within 2-hour sessions (n=13).

Outcome	Participant-reported outcome measures				Physical mobility and exertion measures		
	Provoked mTBI^a^ signs or symptoms, n (%)	Complete without or minimal assist, n (%)	Complete with assist, n (%)	Unable to complete, n (%)	Safely completed, no provoked mTBI signs or symptoms, n (%)	Completed safely, ended test due to other physical reason, n (%)	Unable to complete, n (%)
Health form	0 (0)	13 (100)	0 (0)	0 (0)	—^b^	—	—
PCSS^c^	0 (0)	13 (100)	0 (0)	0 (0)	—	—	—
PSFS^d^	0 (0)	11 (85)	2 (15)	0 (0)	—	—	—
MSQ^e^	—	—	—	—	12 (95)	1 (8)	0 (0)
BCBS-M^f^	—	—	—	—	13 (100)	10 (77)	0 (0)

a mTBI: mild traumatic brain injury.

b Not applicable.

c PCSS: Post Concussion Symptom Scale.

d PSFS: Patient Specific Functional Scale.

e MSQ: Motion Sensitivity Quotient.

f BCBT-M: Buffalo Concussion Bike Test-Modified.

#### Emergent Thematic Analysis

The second aim was an emergent thematic analysis focused on research procedures, participant interaction, physical space, and data collection with consideration to older adults with and without mTBI. We relied on the ICF contextual domains to support the research-related themes of the study protocol and study environment. The theme of the study protocol included categories of time, communication, and logistics. The theme of the study environment included categories of documentation, assessment, and safety. Some of the categories had various perspectives to consider, equally important in considerations derived from both the participant (P) and investigator (I) perspectives (Table 2). Examples of the codes under only the study protocol referred to communication modifications for individual participants and investigators skilled in participant safety assessment. Examples of the codes under the study environment related to skilled investigators who modified equipment to optimize size and fit and mitigate safety risks. These findings were important considerations for conducting future research. For example, investigators may need to consider the physical test environment, equipment adjustability, communication options, and monitored participant assessment (licensed health care providers as investigators) for safe and optimal research study procedures and data collection. Hume et al [17] reported that older adults with mTBI required specific recovery considerations and highlighted limitations in research involving this population [17]. The limitations included recruiting individuals aged >65 years and accounting for age-related psychological and biological differences. Elias et al [11] described that older adults have unique psychological, social, and biological characteristics that have not been developed in the mTBI research to effectively translate into postinjury mTBI management. Although the preliminary findings of this study required further development, the results provided insight into safe and accurate research considerations for older adults with and without mTBI.

This study supported unanticipated contextual relevance within the thematic analysis from the code of “unsolicited participant feedback.” Because the feedback was unexpected, we had no established formal data collection methodology before testing. Surprisingly, the participants (n=10) organically stated positive comments after data collection (Table 2). We interpreted the participants’ responses as novel reflections of their societal role within the framework of the Role Theory in Active Aging [29,30]. The participant feedback findings (Table 2) suggested that participating in research may have meaning to the participants (Figure 1). The participants emoted responses that reflected self-worth, a sense of belonging to a societal role, and successful participation in a social role [29]. These early findings needed further development to support the benefits of older adults participating in research and how this may impact their perceived role in active aging. By identifying and validating positive psychological and functional outcomes associated with research involvement, future studies can help promote broader inclusion of older adults in scientific inquiry. This, in turn, may enhance generalizability and empower older adults to view themselves as active contributors to knowledge generation and societal advancement, supporting healthier, more engaged aging trajectories [32].

### Limitations

This study was a feasibility study in evaluating older adults with and without mTBI and therefore had inherent limitations. The sample size was small, the enrollment was a sample of convenience, and the shortened recruitment timeline was based on resource availability. Although we recruited from demographically different areas and worked with senior services across local facilities, the participants were not representative of the US population. We have discussed more rigorous recruitment and enrollment strategies for improved representation in future research.

Another limitation was having 1 investigator collect data. Early in the study, the investigators established that to ensure participant safety, data collection must be performed by a licensed health care provider with experience in the assessment of older adults. The PI (CAB) addressed mitigating bias by member checking documented participant feedback and deidentifying all data before analysis. Future studies would include training specific to older adults with mTBI and extend data collection to multiple sites.

Diffusion in the study design was also a concern, as some of the participants arrived at the testing site in pairs. The PI (CAB) separated the participants during testing to maintain data collection integrity; however, this was verbalized by the participants as challenging. The participants who had more comorbidities relied heavily on their partners for accuracy in reporting timelines. The PI (CAB) encouraged participants to independently answer to their best ability. Future studies may involve acquiring electronic medical records to triangulate the accuracy of health form reporting. Despite this, the participants were able to complete the health forms without or with minimal cues (N=13, 100%), and this simulated individual participant reporting in a clinic appointment.

### Conclusion

The evaluation of individuals for baseline assessment and initiation of treatment has been identified as crucial in reducing recovery times following mTBI [13-15]. The evaluation of older adults with mTBI, clinical guideline recommendations, and testing tolerance has been limited in the research [11,13]. This feasibility study was critical for further development of methodology and implementation of future large sample studies using provocation testing in older adults with mTBI. This study contributed to a deeper understanding of key contextual variables, including temporal constraints, safety protocols, communication strategies, and environmental settings, that shape research methodologies for evaluating older adults. The study also highlighted the potential benefits of research participation for older adults, particularly with societal expectations surrounding active aging. Further development and validation of these findings could guide physical therapists in delivering safe, evidence-informed care to the aging population with mTBI. Continued research is essential to establish standardized testing protocols that are tailored to the unique needs and vulnerabilities of older adults with mTBI [13].

## Supplementary material

10.2196/76799 Checklist 1 CONSORT 2010 checklist.

10.2196/76799 Checklist 1 Mixed Methods Reporting checklist.

## Abbreviations

BCBT-M: Buffalo Concussion Bike Test-Modified
BPPV: benign paroxysmal positional vertigo
ICF: International Classification of Function, Disability, and Health
IRB: institutional review board
MSQ: Motion Sensitivity Quotient
mTBI: mild traumatic brain injury
PCSS: Post Concussion Symptom Scale
PI: primary investigator
PSFS: Patient-Specific Function Scale
TBI: traumatic brain injury
WMU: Western Michigan University
