# Supplementary material for: Feasibility of Physical Therapy Evaluation Symptom Provocation Tests in Older Adults With Mild Traumatic Brain Injury: Mixed Methods Study
Source: JMIR Form Res. 2025 Oct 23;9:e76799. doi: 10.2196/76799 (PMC12548967; doi:10.2196/76799)
Supplement: Checklist 1 [file formative-v9-e76799-s002.pdf]

Mixed Methods Reporting in Rehabilitation & Health Sciences (MMR-RHS)

Instructions: The following checklist outlines essential information for mixed methods reporting. **1) Indicate "Y" if the standard is *fully met* or "N" and additional comments if lacking**. **2) Document page number where element is located**.

For more specific guidance, refer to the following linked checklists and individual journal requirements: Clinical Trials (CONSORT), Diagnostic Studies (STARD), Measurement Evaluation (COSMIN); Observational studies (STROBE); Intervention (TIDieR); Quality Improvement (SQUIRE); Qualitative (SRQR).

Title	Y/N; Comments
Concisely describes the topic of the study identifying the study as mixed methods	Y
**Abstract**	**Y/N; Comments**
Summarizes key elements using *journal specific* abstract format; For example: Introduction, Methods, Results, Discussion, and Significance/potential impact to rehabilitation and/or societal health	Y; Background, Objective, Methods, Results, Conclusion (Clinical Relevance)
	**Y/N; Page # Comments**
**Introduction**	
Includes literature review on the topic of interest (quantitative, qualitative, and mixed)	Y
Identifies gap that justifies the need for mixed methods approach	Y; however due to limited research on this topic overall, very little mixed methods research was available specific to mTBI and older adults in evaluation and management.
Clearly states overarching goal of the study that supports a mixed methods approach	Y

States the rationale for using mixed methods research	Y; rationale was explained through the need for exploring findings within mixed methods research by qualitative (patient reported symptom response, difficulty in self-care, and quality of life) and quantitative (outcomes measure in formal test measures) literature support.
Clearly identifies discrete aim(s) for qualitative and quantitative components<br>Aims align with corresponding component methods	Y
Provides statement of significance and potential impact	Y
**Methods**	
**Design –** *Clearly describes the mixed methods design* (exploratory sequential, explanatory sequential, concurrent, etc.) used to accomplish the overarching goal of the project:<br>• *Emphasis noted* (i.e., Sequential QUAL--> quan or QUAN--> qual; Concurrent QUAL + QUAN)<br>• *Visual display of overall design highlighting integration* (e.g., model, flow chart, figure)	Y

© 2023 Tovin M. & Wormley M.

Mixed Methods Reporting in Rehabilitation & Health Sciences (MMR-RHS)

Methods (continued…)	Y/N; Page # Comments
Describes and supports the qualitative and quantitative methodologies (phenomenology, randomized control trial) used to accomplish the discrete aim(s) of the project	Y; each aim supported for qualitative versus quantitative designs
States researcher(s) background and contributions to project (e.g. content or methods expertise, relationships to participants)	Y
Identifies setting (e.g. hospital system, geographical location)	Y
**Subjects/Participants** - Clearly describes and supports the following:<br>• Sampling and recruitment<br>• Inclusion/Exclusion criteria<br>• Ethical considerations (consent process, researcher relationship with participants)	Y
**Data collection** - Clearly describes and supports the following:<br>• Pilot study (if applicable)<br>• Instrumentation (validity, reliability)<br>• Implementation matrix (e.g. data source, timeline, type, anticipated outcomes)	Y; feasibility study and instrumentation or clinometric research listed under methodology
**Data analysis** - Clearly states and describes analysis procedures for:<br>• Qualitative<br>• Quantitative<br>• *Mixed Methods (integration)*	Y
**Methodological Rigor** – Clearly describes steps taken to establish rigor:<br>• Qualitative (e.g. credibility, dependability, confirmability, transferability)<br>• Quantitative (e.g. validity, reliability, generalizability)<br>• *Mixed Methods* (validity or legitimacy)	Y; qualitative (examples, credibility and confirmability described in member checking and de-dentification in the cohort establishing themes, categories, codes); Quantitative- not generalizable

	due to feasibility study and small sample size
**Results/Findings**	
Clearly presents findings for study components:<br>• Qualitative (includes data exemplars)<br>• Quantitative<br>• *Mixed Methods-Provides integrated findings/overall study results* (e.g., joint display)	Y; Tables for each and verbal highlights in results<br><br>N; no joint display was achieved due to the limited sample number and unable to infer, however we were able to apply to an established model of active role of aging.
**Discussion**	
Incorporates discussion on implications of integrated findings	Y – although not as robust due to the nature of the feasibility study (inference and causation, not appropriate)
Provides synthesis and interpretation of findings in the context of existing literature and theoretical/conceptual framework	Y- similarly, although not as robust as traditional integrated findings due to the nature of the feasibility study (inference and causation, not appropriate)
Includes subsection of limitations	Y

© 2023 Tovin M. & Wormley M.
